# A randomised controlled trial of early insulin therapy in very low birth weight infants, "NIRTURE" (neonatal insulin replacement therapy in Europe)

**DOI:** 10.1186/1471-2431-7-29

**Published:** 2007-08-10

**Authors:** Kathryn Beardsall, Sophie Vanhaesebrouck, Amanda L Ogilvy-Stuart, Jag S Ahluwalia, Christine Vanhole, Christopher Palmer, Paula Midgley, Mike Thompson, Luc Cornette, Mirjam Weissenbruch, Marta Thio, Francis de Zegher, David Dunger

**Affiliations:** 1 Department of Paediatrics, University of Cambridge, Addenbrooke's Hospital NHS Trust, Hills Road, Cambridge, CB2 2QQ, UK; 2 Neonatal Unit, Rosie Hospital, Hills Road, Cambridge, CB2 2QQ, UK; 3 Neonatal Unit, Kindergeneeskunde, UZ, Herestraat 49, B3000, Leuven, Belgium; 4 Centre for Applied Medical Statistics, Department of Public Health and Primary Care, University of Cambridge, UK; 5 Simpson Centre for Reproductive Health, Royal Infirmary of Edinburgh, 51 Little France Crescent, Edinburgh, EH16 4SA, UK; 6 Department of Paediatrics, Luton and Dunstable Hospital, Lewsey Road, Luton, LU4 0DZ, UK; 7 Neonatal Unit, Leeds General Infirmary, Great George Street, Leeds, LS1 3EX, UK; 8 Neonatal Unit, VU University Centre, Amsterdam, The Netherlands; 9 Neonatal Unit, Hospital Universitari, Passeig Sant Joan de Deu, number 2, 08950 Esplugues-Barcelona, Spain

## Abstract

**Background:**

Studies in adult intensive care have highlighted the importance of insulin and improved glucose control on survival, with 32% reduction in mortality, 22% reduction in intensive care stay and halving of the incidence of bacteraemia. Very low birth weight infants requiring intensive care also have relative insulin deficiency often leading to hyperglycaemia during the first week of life. The physiological influences on insulin secretion and sensitivity, and the potential importance of glucose control at this time are not well established. However there is increasing evidence that the early postnatal period is critical for pancreatic development. At this time a complex set of signals appears to influence pancreatic development and β cell survival. This has implications both in terms of acute glucose control but also relative insulin deficiency is likely to play a role in poor postnatal growth, which has been associated with later motor and cognitive impairment, and fewer β cells are linked to risk of type 2 diabetes later in life.

**Methods:**

A multi-centre, randomised controlled trial of early insulin replacement in very low birth weight babies (VLBW, birth weight < 1500 g). 500 infants will be recruited from 10 centres in the UK and Europe. Babies will be randomised to receive a continuous insulin infusion (0.05 units/kg/h) or to receive standard neonatal care from the first day of life and for the next 7 days. If blood glucose (BG) levels fall infants will receive 20% dextrose titrated to maintain normoglycaemia (4–8 mmol/l). If BG is consistently above 10 mmol/l babies will receive standard treatment with additional insulin infusion. The primary end point will be mortality on or before expected date of delivery, secondary end points will be markers of morbidity and include episodes of sepsis, severity of retinopathy, chronic lung disease and growth.

**Trial Registration:**

Current Controlled Trials ISRCTN78428828. EUDRACT Number 2004-002170-34

## Background

Perinatal survival in very low birth weight infants (less than 1.5 kg) has increased markedly over the last twenty years with improvements in neonatal intensive care [1]. However mortality rates are as high as 20% in those born less than 1 kg, death may be related to either infection or necrotising enterocolitis. Reducing mortality is an important goal of therapy but it is essential that this be achieved without increasing long term morbidity. Post-natal growth may be sub-optimal particularly with regard to head circumference [2] and this can be associated with neuro-psychological problems particularly in the very low birth weight infant [3], and retinopathy of prematurity is still a major problem [4].

Studies of adult intensive care patients have highlighted the importance of blood glucose control on rates of sepsis and survival [5]. In the study reported by Van den Berg, where insulin was used to tightly control blood glucose a reduction in intensive care mortality of 32% (p < 0.04), a reduction in mean ICU stay by 22% (p = 0.005) and a halving of the incidence of bacteraemia (p = 0.003) was observed [6]. Studies in both diabetic and non diabetic patients post myocardial infarction indicate that the use of insulin has not only immediate effects with respect of glucose control but also improves long term outcome [7]. In adults receiving parenteral nutrition insulin has been shown to improve net leucine balance [8]. It is not always clear from these studies whether it is the reductions in blood glucose per se or the anabolic effects of insulin that are important [5]. Furthermore the medical conditions encountered in adult intensive care are not the same as those seen in neonatal units, but nevertheless there are some parallels that suggest that insulin could also have a role in newborn care.

The incidence of hyperglycaemia in premature neonates admitted to intensive care has been reported to be between 20% and 86% [9]; those who are small for gestational age having the greater risk [9]. We have assessed blood glucose control over the first week of life in 8 very low birth weight babies using the *MiniMed *subcutaneous sensor. In these infants receiving standard neonatal care 38% of the readings were more than 10 mmol/l and only 0.5% of the time were readings under 2.6 mmol/l. The hyperglycaemia was particularly common on day 2 and 3 after birth. Many of these babies were given insulin treatment by intravenous infusion, as is standard neonatal practice, when blood glucose levels were higher than10 mmol/l. Hyperglycaemia can lead to significant osmotic diuresis and hence electrolyte imbalance and has been associated with increased risk of intraventricular haemorrhage. Uncontrolled hyperglycaemia has also been associated with increased episodes of sepsis in post surgical and burns patients [10-13]. The premature neonate is relatively immunocompromised and with immature skin and renal function is at increased risk of infection and problems with fluid and electrolyte balance. There is increasing data documenting the association of hyperglycemia in the neonatal population with increased morbidity and mortality [14-17]. Current neonatal practice of intermittent insulin treatment is only partially effective at controlling hyperglycaemia in the infants who require intensive care.

The reason for these prolonged periods of hyperglycaemia is not clear. During fetal life, insulin secretion is largely determined by glucose flux across the placenta and blood samples taken from foetuses at cordocentesis demonstrated an exponential increase in fetal plasma insulin with increasing gestation [18]. Lower plasma glucose and insulin as well as higher glucose insulin ratios have been shown in growth retarded foetuses compared to those who are appropriately grown [18]. At birth, the disruption of placental supply of nutrients leads to a period of catabolism with weight loss being maximal two to three days after birth, birth weight not being recovered often until the age of 7 days. Insulin levels are low during this period only increasing with the establishment of oral feeds and the coupling of insulin secretion to meal-related nutrient and hormonal signals [19-21]. Blood glucose levels during this period of catabolism are maintained by gluconeogenesis and glycolysis driven by catecholamines, growth hormone and cortisol. In a setting of continuing limited substrate availability, these hormones may also play a role by reducing insulin sensitivity, increasing lipolysis, and hence diverting glucose utilisation from muscles to the brain, protecting the fetal brain from the risk of hypoglycaemia [22,23].

In the very low birth weight infant, the period of neonatal catabolism may be much more prolonged, as it may not be possible to initiate oral feeding and thus induce normal insulin secretion. As a consequence, birth weight is often not regained for several weeks. The very high blood glucose levels seen on day 3 or 4 may reflect insulin resistance secondary to high levels of growth hormone and the stress related hormones catecholamines and cortisol. They could also reflect relative insulin deficiency and a failure to compensate for any increasing insulin resistance. Fetal growth restriction may be associated with impaired pancreatic development and a reduced β-cell mass [24-27] although intravenous alimentation is often started soon after birth in these infants, enteral feeding and normal β cell function may not be achieved for several weeks.

Although insulin therapy is used intermittently on the neonatal unit, there are few studies which have formally assessed its benefits [28-34]. These studies have been small and some have compared the use of insulin with reduction in glucose infusion. One study in premature infants using continuous insulin infusions to control hyperglycaemia showed better glucose tolerance with improved weight gain combined with a reduced incidence of sepsis [28]. The use of stable isotopes has demonstrated a role for insulin infusions in reducing proteolysis in the low birth weight infants [31]. Thus potentially, insulin could limit proteolysis and improve weight gain and growth.

In a recent pilot study, we demonstrated that early continuous insulin infusion effectively controlled blood glucose levels over the first week of life and prevented the high blood sugars seen on day 2 or 3 in infants given standard intermittent insulin therapy. These data indicate that insulin replacement early from birth may prevent some of the catabolism and insulin resistance normally observed in the pre-term infant. Thus insulin replacement in the newborn could reverse the risk associated with high blood sugar: it could improve anabolism and weight gain, and theoretically by reducing hyperglycaemia, reduce risk of sepsis. Insulin therapy in the newborn could also have more far reaching benefits. Levels of insulin like growth factor 1 (IGF-1) and the inhibitory IGF binding protein IGFBP-1 are regulated by insulin in the newborn [35-37]. IGF-I has an important role in fetal and postnatal brain growth [38,39]. Furthermore, low IGF-1 levels [40-42] and hyperglycemia [43] have been implicated in the pathogenesis of retinopathy of prematurity. Thus theoretically, improved insulin delivery and restoration of IGF-I levels could have important implications for the long term outcome as well as the short term survival of very low birth weight babies.

### Hypothesis

We propose that relative insulin deficiency in the very low birth weight baby leads to catabolism, insulin resistance and hyperglycaemia during the first week of life. High blood glucose levels may lead to osmotic diuresis and increase the risk of sepsis. Insulin deficiency may contribute to slow weight gain and impaired IGF-I generation which could have implications for risk of retinopathy, brain growth and later neurodevelopmental outcomes. We hypothesise that early intervention with continuous insulin replacement will prevent catabolism and improve glucose control, and could reduce neonatal morbidity and mortality.

### Aims of the study

To carry out a multi-centre, randomised controlled study of early insulin replacement in very low birth weight infants (VLBW < 1500 g). 500 children will be randomised to receive either a continuous infusion of insulin (0.05 u/kg/hr) from within 24 hours of birth and for the first 7 days of life, or to act as controls and receive standard neonatal care.

### Objectives

#### Primary

To investigate whether an early fixed dose insulin infusion, combined with variable dextrose support to maintain normoglycaemia, will reduce mortality on or before expected date of delivery in the very low birth weight neonate.

#### Secondary

(i) incidence of sepsis in the first 2 weeks of life; (ii) growth at 28 days; (iii) incidence of necrotizing enterocolitis (iv) severity of retinopathy of prematurity; (v) incidence of intracranial haemorrhage (vi) incidence of chronic lung disease (vii) mortality before 28 days of age (viii) total number of days in neonatal intensive care prior to discharge home

#### Efficacy outcomes

(i) Blood glucose control over the first week of life as assessed by the *MiniMed *subcutaneous continuous glucose monitor (CGMS); (ii) Effects of treatment on circulating IGF-I, IGFBP-1, cytokines.

## Methods/Design

### Study design

The study is a multicentre randomised controlled trial. It is based in 10 neonatal intensive care units and 500 babies will be recruited over 2 years. Very low birth weight babies (VLBW < 1500 g) will be recruited within 24 hours of delivery and followed until expected date of delivery (40 weeks). They will be randomised to either treatment with early fixed dose insulin (0.05 unit/kg/hr) with 20% dextrose to maintain normoglycaemia, or to receive standard neonatal care. Those randomised to treatment will receive a fixed dose of insulin combined with variable 20% dextrose support throughout the first week. Additional insulin will be infused if blood glucose levels are consistently above 10 mmol/l; and an infusion of 20% dextrose will be started if blood glucose falls to <4 mmol/l to prevent hypoglycaemia (blood glucose < 2.6 mmol/l). Controls will receive standard neonatal care. All babies will be monitored using a *MiniMed *continuous glucose monitor for 7 completed days and have a blood sample taken on Study days 1,3, 7 and 28. A urine sample will be collected from all babies on Study day 7 and all babies will have details of their clinical care recorded daily in the first week then weekly until 4 weeks, at 36 weeks corrected age, at expected date of delivery (40 weeks) and at discharge home.

### Study population

#### Infant Selection

Infants must fulfill all inclusion criteria – birth weight < 1500 g, requiring intensive care and in whom it is considered appropriate to continue intensive care, less than 24 hours of age, and written informed parental consent. Infants must not have any of the following exclusion criteria: maternal diabetes including gestational diabetes, babies where the appropriateness of continuing intensive care is being discussed, or major congenital anomalies

### Inclusion in the study

If all the inclusion and exclusion criteria are met the patient will be included in the study and allocated a sequential patient number. The randomisation will be stratified by centre, by birth weight (<1000 g, 1000–1500 g), and by gestational age (<25 weeks and ≥25 weeks). A 24 hour internet based randomisation programme will be used which requires user name and is password secure. Treatment with insulin will begin as soon after birth as is possible following randomisation. Patient data will be analysed on the basis of intention to treat. Consent will be taken by a health care professional who has a good working knowledge of the aims and practicalities of the study. Parents will be given a written information leaflet and given the opportunity to read and discuss the information they have been given alone. As patients need to be recruited within 24 hours of delivery, a system of continuing consent will be used over the first 3 days to ensure that parents are happy with the decision to consent to their baby's participation in the study.

### Study medication

#### Study product

Insulin aspart (*NovoNordisk*) (pyr) for intravenous injection. Insulin will be given intravenously at a fixed rate of 0.05 u/kg/hour. It will be prepared as a standard strength solution of 25 units/kg insulin aspart in 50 mls of 0.9% sodium chloride to run at 0.1 ml/hr, equivalent to 0.05 u/kg/hour. This will be combined with a variable rate 20% dextrose infusion when required to maintain normoglycaemia. Infusion dose will be based on the infants birth weight and adjusted only if there is concern that the recorded birth weight was inaccurate

### Study device MiniMed CGMS (continuous glucose monitoring sensors)

These comprise a disposable glucose oxidase-based platinum electrode sensor that catalyzes interstitial glucose generating an electrical current every 10 seconds, which is recorded via a cable by a pager sized monitor (6 × 9 × 2 cm). The monitor records average values every 5 minutes, giving a total of 288 readings per day. Glucose values outside the range 2.2–24 mmol/l (40–430 mg/dl) are reported as <2.2 mmol/l (<40 mg/dl) or >24 mmol/l (>430 mg/dl) respectively. Nursing and medical staff will be instructed in the use of the monitor and asked to enter all blood glucose (BG) measurements taken for clinical reasons using near patient monitoring devices into the monitor for calibration. The data cannot be viewed in real time and therefore cannot impact on clinical care.

### A. Babies randomised to insulin treatment

As is usual practice initial management will involve obtaining intravenous access, measuring blood glucose and initiating a dextrose infusion.

*Study babies will in addition require: *i) Insertion of a *MiniMed *sensor (subcutaneous insertion in the thigh). ii) Preparation of *fixed dose insulin infusion *iii) Preparation of a separate 20% dextrose infusion, for use if the BG should fall to <4.0 mmol/l to prevent hypoglycaemia.

#### i) Insertion of a MiniMed sensor (subcutaneous insertion in the thigh)

The sensor will be inserted subcutaneously in the thigh. It will be secured in place with a clear occlusive dressing. The sensor will be sited in the lateral aspect of the thigh by a member of the study team who has been appropriately trained. The sensor will be sited using aseptic technique. The legs will be examined to ensure an area of unbroken skin over the lateral aspect of the thigh. The skin will be gently cleaned with damp sterile gauze and allowed to dry. If the skin is very friable then a fine spray of *cavilon *will be applied to form a barrier. Any adhesive will be trimmed to ensure minimal contact with the skin but allowing for secure attachment. The individual inserting the sensor will document clearly the time and site of sensor insertion and condition of the site. All BG levels will be entered into the *MiniMed *Sensor to allow for calibration. These will be taken as frequently as clinically indicated but a minimum of 4 readings (approximately 6 hourly) must be entered every day. These will be entered within 5 minutes, as the monitor will record subcutaneous readings every 5 minutes. If there are problems with dislodgement of the sensor it can be replaced on up to two occasions. The sensor should be left in situ for 12 hours after completion of the insulin infusion and should therefore be removed at midday on Day 8.

#### ii) Fixed Dose Insulin Infusion

*Treatment: 25 units/kg insulin (aspart) in 50 mls of 0.9% (or 0.45%) sodium chloride to run at 0.1 ml/hr equivalent to 0.05 u/kg/hour. *The insulin solution should be prepared in a 50 ml volume to allow a minimum of 20 mls of the solution to be flushed through the connection tubing. This is to reduce the adsorption of further insulin to the plastic tubing during infusion.

#### iii) 20% dextrose

All babies receiving insulin will have a 20% dextrose infusion prepared for infusion if the blood glucose levels fall <4.0 mmol/to prevent hypoglycaemia. Calculation of the dose of insulin will be based on the baby's birth weight and only changed if this is felt in retrospect to be inaccurate.

#### Initiating treatment

If the blood glucose level is >4.0 mmol/l and the baby is receiving a minimum glucose infusion of 4 mg/kg/min (60 ml/kg/d of 10% dextrose) then the *fixed dose insulin infusion *regimen will be started. If the blood glucose is <4.0 mmol/l then 20% dextrose will be infused and blood glucose rechecked within one hour, repeatedly increasing 20% dextrose and checking BG hourly until BG is >4.0 mmol/l. Only when the BG is >4.0 mmol/l will the fixed dose insulin infusion be started. Once the insulin infusion has started the BG will be checked hourly until stable and then 2 hourly increasing to a time interval of 4 hours. If a baby is stable the time interval may be increased to a maximum of 6 hours under the guidance of the Principal Investigator. The fixed dose insulin infusion will start within 24 hours of delivery and will run for seven completed days. The fixed dose insulin infusion will be infused via the same line that the baby is receiving its main source of dextrose/parenteral nutrition. This will ensure that if there is a break in the delivery of the main source of dextrose to a baby that there will also be a simultaneous break in the infusion of insulin.

The *fixed dose insulin infusion *will be maintained but any decisions regarding a baby's maintenance glucose infusions and fluid requirements will be taken by the medical team caring for the baby. If there are any clinical changes in the rate of glucose being infused or a clinical insulin infusion is started then a blood glucose will be rechecked hourly until stable.

#### Maintaining 'Normoglycaemia' (BG 4–8 mmol/l) (Figure 1)

**Figure 1:**
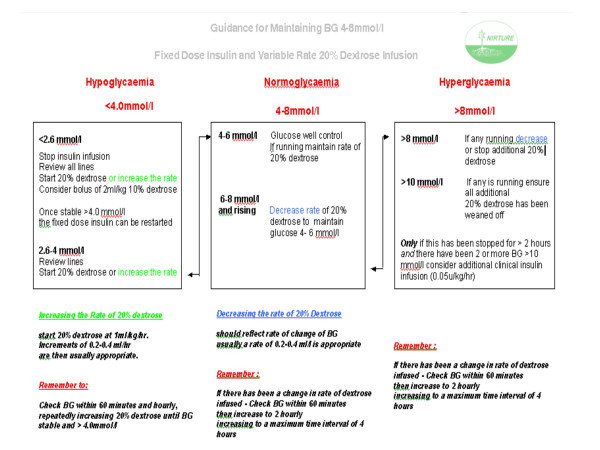
Guidance for maintaining blood glucose using fixed dose insulin and variable rate 20% dextrose.

Aim to maintain blood glucose 4–8 mmol/l using the infusion algorithm shown in Figure 1.

##### Hyperglycaemia

If blood glucose levels rise (>10.0 mmol/l on two occasions) and there is no additional 20% dextrose being infused treatment with insulin should be started to maintain normoglycaemia (BG 4–8 mmol/l).

##### Hypoglycaemia

If the blood glucose level falls <4.0 mmol/l, intravenous lines should be reviewed and additional 20% dextrose infused to avoid hypoglycaemia – starting at 1 ml/kg/hour extra (see Figure 1). A review should be made of the amount of dextrose being received and the additional 20% dextrose infusion that has been prepared should be titrated to prevent hypoglycaemia. Repeated BG measurements should be taken hourly repeatedly increasing the 20% dextrose until the value is stable and >4.0 mmol/l. This should prevent BG levels falling to <2.6 mmol/l. However if BG < 2.6 mmol/l then the insulin infusion should be stopped, all lines checked to ensure the baby is receiving the dextrose prescribed and additional 20% dextrose infused. A repeat BG should be taken after 60 minutes, and hourly with the 20% dextrose repeatedly increased to maintain the BG 4.0–8 mmol/l. Close liaison with the medical and nursing staff is critical to make sure they understand the treatment algorithm and that the study is aiming to control the BG between 4–8 mmol/l. They must be aware of the importance of taking regular and frequent BG measurements to ensure euglycaemia and that 20% dextrose should be started if a BG level is recorded that is <4.0 mmol/l.

##### Stopping Treatment

After the baby has received 7 days of insulin the infusion should be stopped at midnight on Study Day 7. If the baby has additional 20% dextrose running as well as the insulin then the insulin should be stopped first and the 20% dextrose weaned of over a period of several hours. In all cases the blood glucose should be monitored closely after stopping the insulin to ensure the baby does not become hypoglycaemic or hyperglycaemic. The Minimed sensor should be left in situ for 12 hours after stopping the insulin and be removed at midday on Study Day 8. Appropriate clinical management should be taken by the clinicians responsible for the baby's care.

### B. Babies randomised to control arm

As is usual practice initial management will involve obtaining intravenous access, measuring blood glucose and initiating a dextrose infusion.

*Study control babies will in addition require*

i) Insertion of a *MiniMed *sensor (subcutaneous insertion in the thigh, as described above).

ii) Blood Glucose control as per standard clinical protocol

Blood glucose control

These babies will receive standard clinical care.

Therefore if BG:

<2.6 mmol/l standard unit management of hypoglycaemia

>10 mmol/l if 2 or more readings are >10 mmol/l consider addition of insulin infusion

Once BG has fallen to <10 mmol/l then any insulin infusions should be stopped.

### Measurement of outcomes

#### Primary outcomes

Death on or before expected date of delivery (taken as date considered the most accurate estimate of delivery date).

#### Secondary outcomes

*i. Episodes of sepsis in the first 2 weeks: *

a) Culture positive systemic infection will be defined as microbiologically positive cultures of blood, cerebrospinal fluid or suprapubic aspirate of urine plus clinical signs of sepsis.

b) Culture negative infection will be defined as clinical signs suggestive of sepsis and considered to warrant >48 hours of antibiotics but with negative cultures.

***ii. Growth: ***All babies will have measurements of weight, length and head circumference at birth and every seven completed days after recruitment until EDD. Growth will be assessed as change in weight, length and head circumference standard deviation score (SDS) from birth to 28 days using conditional charts.

***iii. Incidence of necrotizing enterocolitis: ***defined as radiological evidence of necrotizing entercolitis assessed by consultant radiologist.

***iv. Retinopathy of prematurity: ***All these babies will be routinely screened, for retinopathy of prematurity and will be graded using the internationally recognised grading system [44,45]. Infants will be classified by the most severe degree of retinopathy.

***v. Incidence of intracranial haemorrhage: ***defined as cranial ultrasound evidence as assessed by Consultant Neonatologist.

***vi. Chronic lung disease: ***defined as respiratory support or oxygen dependency at 36 weeks corrected gestational age [46].

*vii. Death within and including the first 28 days after delivery*

***viii. Days of neonatal intensive care***: As defined by BAPM 2002[47].

#### Efficacy

***i. Percent of time normoglycaemic: ***All babies will have a continuous glucose sensor *in situ *for the 7 completed days. Sensors will be inserted within 24 hours of birth and left in situ for 7 days from the time of insertion. The sensors will be removed 12 hours after the insulin infusion has stopped. This will take a measure of interstitial glucose every 5 minutes and will give an assessment of glucose control throughout the first week. The percent of time spent with blood glucose <2.6 mmol/l, 2.6–10.0 mmol/l, >10 mmol/l and <4.0, 4.0–8.0 mmol/l, >8.0 mmol/l will be measured.

***ii. IGF-I and IGFBP-1: ***measured in blood taken on Study Days 1, 3, 7 and 28.

**iii) *cytokines: ***measured in blood taken on Study Days 1, 3, 7 and 28.

**iv) *protein catabolism: ***measured by urinary 3-methylhistidine/creatinine ratio on Study day 7.

### Continuation of insulin treatment after 7 days

If a baby becomes hyperglycaemic after the study dose of insulin has been stopped it will be a decision of the doctors responsible for the patients medical care whether insulin should be restarted, what preparation will be used and what dose administered.

### Concomitant therapy

All other therapy considered necessary for the patients welfare may be given at the discretion of the medical staff in charge of the infant's medical care.

### Withdrawal from treatment

A patient will be withdrawn from the study if in the opinion of the Investigator it is medically necessary or if it is the parents wish for a child to be withdrawn. The reason for withdrawal should be clearly documented in the Case Report Form and reported to the co-ordinating centre within 24 hours.

### Follow up

Contact details and hospital identifiers for all patients in all centres will also be collected so that future follow up will be possible. If there are effects on short term growth it will be important to review growth, body composition and insulin resistance in later childhood, as well as neurodevelopmental outcome. As is current practice in early neonatal deaths, in any baby who dies during the study period, the parents would be approached for consent to a post mortem being performed.

### Statistics

Intention to treat analyses will be performed comparing the outcome of all infants allocated to early insulin treatment compared to those allocated placebo, regardless of how complete treatment was. Statistical analyses will use standard methods to calculate the event rates, time-to-event rates, relative risks, and numbers needed to treat for each outcome in the treatment compared with placebo group, along with 95% confidence intervals. Where appropriate χ2, Fisher's exact or log rank tests of significance will be performed and presented with p-values.

The primary outcome will be modelled with logistic regression in order to evaluate a 95% confidence interval for odds ratio according to treatment group adjusted for key prognostic variables, including if significant, geographic centre. No formal interim analyses are intended although the trial's Data Monitoring Committee (DMC) will meet regularly, have access to unblinded data and would recommend early termination to the Trial Steering Committee ifjudged appropriate based on ethical, safety or efficacy considerations. Time to event analyses stratified by category of birthweight, using Kaplan-Meier survival analyses and stratified logrank tests will be used to assist DMC decision-making. For the secondary outcomes, modelling with either multiple regression (for continuous variables) or logistic regression (for binary variables) will be employed, adjusting for the same baseline covariates as the primary analysis. If individual terms in models are non-significant at level 0.05, then simpler models may be presented instead.

A secondary analysis will be on an "as treated" basis, defining treatment exposure as at least 4 days without interruptions of more than 1 hour.

### Power calculation

Sample size is based on the primary outcome variable of mortality. This is based on statistical power analyses for mortality of 20% by EDD. To predict an absolute difference of 10% with 80% power at the 5% level of significance we would need 428 patients. Non-compliance is not likely to be a problem, although withdrawal from treatment may be 5–10%. Thus we aim to recruit 500 patients. Each unit has an admission rate of approximately 100 very low birth weight babies per year. Due to the confined period of recruitment being within 24 hours of delivery we predict 50% availability for recruitment (some babies will be transferred in after delivery). Coupled with an estimated 60% rate of consent we predict we would recruit 500 infants over a 2 year period with a realistic safety margin. For the primary outcome measure of mortality by EDD loss to follow up should be negligible.

### Ethical and regulatory issues

#### Patient information and consent

Parents will be given full verbal and written information regarding the objective and procedures of the study and the possible risks involved. If possible parents will be approached prior to delivery to allow time for considering study details prior to giving assent. However all parents would be asked to confirm informed consent in writing after a baby's birth. As patients need to be recruited within 24 hours of delivery, a system of continuing consent will be used over the first 3 days to ensure that parents are happy with the decision to consent to their baby's participation in the study.

### Regulatory bodies

EUDRACT Number: 2004-002170-34

MHRA: DDX granted 21/4/04 became a CTA 1/4/04

UK Sponsor Cambridge University Hospital NHS Foundation Trust

### Study timetable

The study started in Cambridge in January 2005 following confirmation of final protocol approval by ethics committees and MHRA regulatory approval. New study centres were then taken on over the subsequent 12 months and recruitment is currently on target to be completed by December 2007. All laboratory analyses will be completed within 6 months of the last patient recruited.

## Discussion

This study is based on the hypothesis that early intervention with insulin replacement prevents catabolism and the hyperglycaemia seen during the first week of life, in the VLBW preterm infant. The strategy is not simply that used in adult intensive care to actively titrate insulin in subjects with hyperglycaemia, although the adult outcome data particularly with respect to reductions in sepsis, with tight glycemic control, may have interesting parallels. Our approach, to administer continuous insulin with glucose cover also provides additional safety compared to sliding scales of insulin generally used in this population, and also highlights the differences with adult ICU protocols. Although mortality and morbidity rates are much lower in infants 1–1.5 kg compared to those <1 Kg it was felt that these infants may well benefit from any long term effect on growth and/or reduction in the incidence of retinopathy and therefore they have been included in the protocol.

## Summary

Very low birth weight infants requiring intensive care have relative insulin deficiency often leading to hyperglycaemia during the first week of life which has been associated with increased mortality and morbidity. The physiological influences on insulin secretion and sensitivity, and the potential importance of interventions to improve glucose control at this time are not well established. However theoretically, improved insulin delivery and restoration of IGF-I levels could have important implications for short term survival as well as the long term outcomes of these VLBW babies. This is a multi-centre, randomised controlled trial of early insulin replacement in VLBW babies. Babies will be randomised to receive a continuous insulin infusion (0.05 units/kg/h) with 20% dextrose support or to receive standard neonatal care for the first 7 days of life. The primary end point will be mortality on or before expected date of delivery, secondary end points will be markers of morbidity and include episodes of sepsis, severity of retinopathy, chronic lung disease and growth.

## Abbreviations

IGF-I insulin like growth factor one

IGFBP-1 insulin like growth factor binding protein one

VLBW very low birth weight

BAPM British Assocoation of Perinatal Medicine

CGMS continuous glucose monitoring sensor

BG blood glucose

DMC Data Monitoring Committee

## Competing interests

The author(s) declare that they have no competing interests.

## Authors' contributions

FdZ, DBD and ALOS conceived of the study and participated in the design of the protocol. KB performed the pilot study and participated in the design of the protocol and drafted the study protocol. ALOS, JSA, CV, PM, MT, LC, MW and MT participated in the design of the study and final protocol.

## Pre-publication history

The pre-publication history for this paper can be accessed here:


